# Practice, disaster preparation training needs, and associated factors in nursing staffs operating at Amhara regional state referral hospitals in Ethiopia

**DOI:** 10.1016/j.heliyon.2022.e10856

**Published:** 2022-10-01

**Authors:** Lehulu Tilahun Yirdaw, Birhanu Dessu Teferra, Mulusew Zeleke Belay, Kirubel Dagnaw Tegegne, Ms Infant Rani Augustin

**Affiliations:** a Wollo University,Department of Emergency and Ophthalmic Health, Dessie, Ethiopia; b Wollo University, Department of Adult Health, Dessie, Ethiopia; c Wollo University, Comprehensive nursing, Dessie, Ethiopia; d Subharti University, Panna, Dhai Maa Sbharti Nursing College, Meerut, India

**Keywords:** Practice, Training need, Disaster preparation

## Abstract

**Background:**

Natural & human-made disasters are occurring at alarming rates around the world, necessitating more training and preparing frontline emergency department nurses.

**Methods:**

The findings were derived from a hospital-based cross-sectional study. The study included all emergency department working nurses from the region's referral institutions. Self-administered written questionnaires were used to collect disaster information from respondents. Epidata software manager v4.6.0.2 was used to enter and code data, which was then exported to spss version 26 for additional analysis.

**Result:**

The majority of our participants were 68-year-old men (66.7 percent). Furthermore, the average age of data respondents was 31.2 ± 5.7. It is discovered that 25 (24.5%) of participants have adequate experience, while 75 (75.5%) of responders have insufficient practice. In addition, 40.9% of responders require training in first aid and treatment concepts, 37.3% require disaster preparedness training, and 31.4% require basic disaster response principles training. In multivariate analysis, training in a hospital setting (P value = 0.047, OR: 0.282, 95 percent CI: (0.081–0.985) and simulation in a hospital setting (P value = 0.002, OR: 0.071, 95 percent CI: (0.055–0.530) were significantly linked with disaster preparedness practice.

**Discussion:**

Levels of disaster practice, training, and their respective associated factors are discussed, along with other findings in the subject.

**Conclusions:**

Because emergency department nurses' disaster preparedness skills are insufficient, training involving drills and simulations, as well as teaching, is required.

**Implications for Nursing and Health policy:**

It aids in effective victim care, rehabilitative services, and emergency and disaster prevention. It may also aid in the priority of care. This will ultimately increase the effectiveness of emergency department care. The research findings may also aid in the establishment of a formal emergency and disaster preparedness framework in emergency departments.

## 1 Introduction

A disaster can be defined as an emergency scenario that puts people's lives in jeopardy [1, 2, 3]. Every day, calamities can strike anywhere in the world, affecting the entire society [2]. The World Health Organization (WHO) pays close attention to this issue and encourages and advises countries to develop their own disaster preparedness plans [3]. This emergency situation could result in property damage as well as human life. As a result, the position of emergency department/ED nurses emerges, necessitating capacity building for these health professionals [4].

Disasters are occurring at alarming rates around the world, necessitating more training and preparing frontline emergency department nurses [2, 5]. This disruptive occurrence has caused visible damage to less developed countries, owing to the connection of disaster impacts with their economy [2]. Around 1.6 million people die each year as a result of disasters. In addition to infectious diseases, the impact becomes a double burden for developing countries [6]. Despite the fact that disasters are controlled and preventable, they remain serious public health concerns. Traumatic injuries, particularly those caused by road traffic accidents, are currently important concerns in Ethiopia [7].

## 2 Materials and methods

A hospital-based cross-sectional study was undertaken at emergency departments of Amhara regional state referral hospitals in March and April 2020G.C. During the study period, all emergency department working nurses at Amhara regional state referral hospitals were deemed to be study subjects.

The survey included all emergency department working nurses at Amhara regional state referral hospitals. Those on study, annual, or maternity leave, on the other hand, were not eligible for this study.

The study included all emergency department working nurses from the region's referral institutions. Self-administered written questionnaires were used to collect disaster information from respondents. From March through April 2020G.C. five data collectors and two supervisors will be available to collect the necessary data.

Data was gathered using the knowledge, attitude, and practice questionnaire and the emergency preparedness information questionnaire/EPIQ, which were adapted from [2] and used in numerous articles.

We conducted training for both data collectors and supervisors in order to preserve the study's quality. In addition, an information questionnaire tool for disaster preparation was used. The Wisconsin Nurses Association suggested that the instrument be used [2]. Pretesting was also conducted on 5% of respondents at Boru Meda Hospital before the actual data collecting began. Before data entry, the supervising primary investigator examined the completed questions.

Epidata software manager v4.6.0.2 was used to enter and code data, which was then exported to spss version 26 for additional analysis. The findings were then presented using tables and figures. Binary logistic regression analysis model was used to examine the relationship between independent and dependent variables. As a result, bi-variable logistic regression and multi- variable regression was performed.

Using the enter technique of variable selection, variables with a P-value of less than 0.25 were chosen and entered into a multi-variable logistic regression. Crude odds ratio/COR, with its 95 percent confidence interval and P-value, were used to make a decision in bi-variable logistic regression. Finally, the adjusted odds ratio/AOR, 95 percent confidence interval, and P-value were calculated to estimate the effect of factors on the outcome of interest after bivariable analysis.

We proceeded only after receiving ethical approval from Wollo University's College of Medicine and Health Sciences' Research and Ethics Review Committee. Following that, permission was obtained from the individual hospital administrations. Finally, written consent was gained from participants, and the study's goal was explained to them, and participation was solely voluntary.

## 3 Results

A total of 115 emergency department working nurses were observed during the study. There were 106 respondents who were eligible for the study, 102 of them had already participated, and data was obtained with a response rate of 96.2 percent due to the non-return of certain tools.

The majority of our participants were 68-year-old men (66.7 percent). Furthermore, the average age of data respondents was 31.2 ± 5.7. The majority of individuals were married (58.8%) or single (41%). (41.2 percent). The majority of data respondents (38.2%) had worked for 3–6 years. The majority of the 82 participants (80.4%) have a bachelor's degree in comprehensive nursing (Table 1).

**Table 1:** Socio demographics on disaster preparation, Emergency Department, Amhara Regional State Referral Hospitals, Ethiopia.

Variables		Frequency	Percent (%)
	Male	68	66.7
	Female	34	33.3
Marital Status	Single	41	40.2
	Married	60	58.8
	Divorced	1	1.0
Clinical Experience	<3 years	14	13.7
	3–6 years	39	38.2
	6–9 years	17	16.7
	>9 years	32	31.4
Highest level of education attained	BSC incomprehensive Nurse	82	80.4
	BSC in ECCN	9	8.8
	MSC in EMCCN	3	2.9
	Others∗	8	7.8

∗ MPH in epidemiology, EMT (Emergency Medical Technician).

According to the study, the majority of the respondents 56 (54.9%) said that catastrophe drills were not conducted in their institution. Only 19 (18.6%) of participants were notified about the disaster drill that took place in their institution. Furthermore, 27 (26.5%) of the interviewees had no idea if their hospital conducted disaster drills or not.

Similarly, the majority of participants 80 (78.4%) said their hospital does not provide continuing catastrophe training, while only 15 (14.7%) said their institution does provide ongoing disaster training. 7 (6.9%) are unsure whether or not disaster training was conducted.

**Figure 1:**
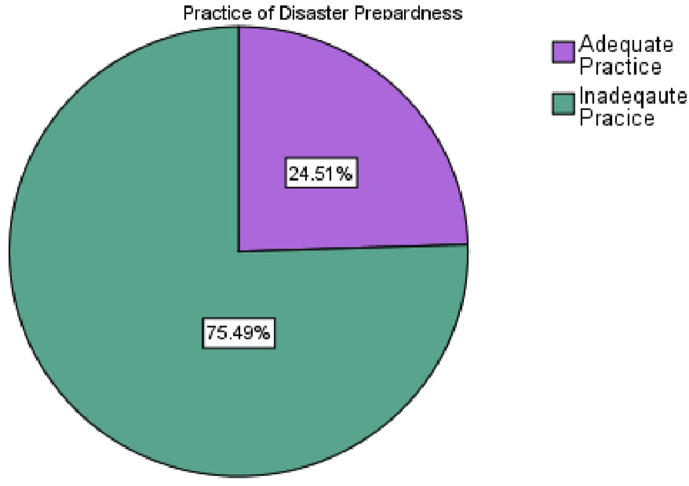
Practice level of participants with regard to disaster preparedness, Emergency Department, Amhara Regional State Referral Hospitals, Ethiopia.

In addition, 67 (65.7 percent) say their disaster plan is not updated on a regular basis, while 19 (18.6%) say their hospital's disaster plan is updated on a regular basis. Furthermore, 16 (15.7 percent) have no knowledge of the issue. Twelve (63.16 percent) of those who said "yes" indicated disaster plans are revised as needed. Others 4(21.05%), 1 (5.26%), and 2 (10.53%) indicated their catastrophe plans are updated every 3–6 months, every 6 months, and every year, respectively. The participants' disaster practice averaged 5.97 ± 1.21. As a result, it is discovered that 25 (24.5%) of participants have appropriate practice, while 75 (75.5%) of respondents have inadequate practice (Table 2 & Figure 1).

**Table 2:** Practice of data respondents with regard to disaster preparation, Emergency Department, Amhara Regional State Referral Hospitals, Ethiopia.

Variables		Frequency	Percent (%)
Are Disaster drills done at your Hospital?	Yes	19	18.6
	No	56	54.9
	Don't know	27	26.5
	Total	102	100.0
Is there ongoing Disaster Training?	Yes	15	14.7
	No	80	78.4
	Don't know	7	6.9
	Total	102	100.0
Does the Emergency Operational (disaster) plan periodically Updated?	Yes	19	18.6
	No	67	65.7
	Don't know	16	15.7
	Total	102	100.0
If Yes how Often?	When need arises	12	63.16
	Every 3–6 months	4	21.05
	Every 6 months	1	5.26
	Every Year	2	10.53
	Total	17	100.00
Mean ± SD = 5.97 ± 1.21

Thirty-one percent (30.4%) of all respondents said they require disaster response training. In addition, 40 percent (39.2%) require first-aid and treatment fundamentals. Rescue and evacuation of wounded, triaging, disaster readiness and response, post-disaster psychological relief, and epidemic prevention were all needed by 21 (20.6 percent), 27 (26.5 percent), 37 (36.3 percent), and 18 (17.6%) participants, respectively. The average demand for disaster preparedness training among respondents was 12.059 ± 2.14 (Table 3).

**Table 3:** Disaster preparation raining need of respondents (n = 102) at Emergency Department, in Amhara Regional State Referral Hospitals, Ethiopia.

Variables		Frequency	Percent (%)
Basic principles of disaster response	Yes	31	30.4
	No	71	69.6
First aid and Treatment principles	Yes	40	39.2
	No	62	60.8
Rescue and transport of the wounded	Yes	21	20.6
	No	81	79.4
On Triaging	Yes	27	26.5
	No	75	73.5
Disaster preparedness and response plans	Yes	37	36.3
	No	65	63.7
Post disaster psychological relief	Yes	18	17.6
	No	84	82.4
Mean ± SD = 12.059 ± 2.14			

A bivariate study revealed that disaster awareness, training in a hospital, and simulation in a hospital setting were all strongly associated with disaster preparedness practice. Practice in disaster preparation is 3 times more likely to be related with disaster awareness than those who are not aware, with a P value of 0.223, OR: 3.354, 95 percent CI: (1.073–10.481). Also, non-hospital training is 85 percent less likely to be connected to disaster preparation practice, with an odds ratio of P value = 0.001, OR: 0.150, 95 percent CI: (0.047–0.482). Simulating in a hospital setting is also 87 percent less likely to be linked with disaster preparation practice, with an odd of P value = 0.000, OR: 0.127, 95 percent CI: (0.043–0.378).

By using multivariate analysis variables that are significantly associated with practice of disaster preparation are training performed in a hospital set-up and simulation done in a hospital. Thus, not performing training in a hospital is 72% less likely to affect practice in disaster preparation with an odd ratio of {P value = 0.047, OR: 0.282, 95%CI: (0.081–0.985)}. In addition, practice of disaster preparedness was 92% less likely associated with no simulation at hospital with an odds of {P value = 0.002, OR: 0.071, 95%CI: (0.055–0.530)} (Table 4).

**Table 4:** Result of risk estimate on practice of disaster preparation at Emergency Department, in Amhara Regional State Referral Hospitals, Ethiopia, 2020.

	Practice of disaster preparation			Odds Ratio			
		Yes	No	P value	COR (95% CI)	P value	AOR (95% CI)
Training Performed in a hospital	Yes	9	6				
	No	16	71	0.001∗	0.150 (0.047–0.482)	0.047∗∗	0.282 (0.081–0.985)
Simulation done at hospital set up	Yes	20	26				
	No	5	51	0.000∗	0.127 (0.043–0.378)	0.002∗∗	0.071 (0.055–0.530)
Having disaster awareness	Yes	7	8	0.037∗	3.354 (1.073–10.481)	0.223	0.370 (0.075–1.832)
	No	18	69				

COR = Crude Odds ratio, AOR = Adjusted Odds Ratio, C.I= Confidence Interval.

∗ Variables significantly associated in bivariate analysis.

∗∗ Variables significantly associated in through multivariate analysis.

## 4 Discussion

This result indicates that 75.5 percent of participants had insufficient disaster preparedness practice. Only 24.5 percent of responders have adequate disaster preparedness practice at the hospital emergency department level, on the other hand. This conclusion is backed by research conducted in Malaysia and Tehran, which indicated that only individuals who have participated in disaster response, education, and training have enough experience. Saudi Arabia also has a skills shortage [8, 9, 10].

According to the findings of the study, 56 percent of respondents said that catastrophe drills were not conducted in their hospital. On the other hand, 80 percent (78.4%) responded that their hospital does not provide continuing disaster training. Similarly, according to research conducted at a Johannesburg hospital, 92.8 percent of nurses indicated that they require disaster ready training. Similarly, other hospital nurses in Johannesburg answered in the same way, indicating that 100 percent of catastrophe abilities are required [9, 11].

We determined that 40 (39.2%) responders need first-aid and treatment concepts training, 37 (36.3%) need disaster preparedness training, and 31 (30.4%) need fundamental disaster response principles training.

The article finds that disaster awareness, hospital training, and hospital simulation are all strongly associated with disaster preparedness practice, according to bivariate analysis. Training in a hospital setting and simulation in a hospital are variables that are substantially associated with disaster preparedness practice, according to multivariate research. This is corroborated by data from Tehran, which show that catastrophe education and training improve professional skill levels [5].

## 5 Conclusion

To summarize, 75.5 percent of the participants have insufficient disaster planning experience. However, only 24.5 percent of respondents have adequate disaster preparation experience at the hospital emergency department level.

We can also deduce that 54.9 percent of hospitals do not conduct disaster exercises. We may conclude that disaster exercises are not conducted in hospitals (only 18.6% of respondents underwent drills). In addition, 78.4% of respondents said their hospital does not provide continuing catastrophe training. Due to continuing catastrophe training, just 14.7 percent of participants responded. Furthermore, 65.7 percent of participants say the catastrophe plan is not updated on a regular basis.

In addition, according to this study, 39.2 percent of referral hospital emergency departments require disaster preparedness training on first aid and treatment principles, 36.3 percent require disaster preparation training, and 30.4 percent require disaster response training on fundamental concepts.

Finally, in bivariate analysis, variables that were substantially linked with disaster preparedness practice were disaster awareness, hospital-based training, and hospital-based simulation. Training in a hospital setting and simulation in a hospital are variables that are substantially associated with disaster preparation practice, according to multivariate research.

## 6 Recommendation

The finding resulted most respondents as having an inadequate skill with regard to disaster preparation; the regional health office to facilitate a skill-based training through dills or simulation to health professional nurses working at emergency and accident departments with regard to disaster preparation.

In addition, in conjunction with health offices and other concerned sectors, the individual regional referral hospital is better positioned to increase short-term and long-term disaster preparedness training for front-line department working professionals. Similarly, emergency department working nurses are encouraged to do their part by doing more studying and improving their disaster preparedness skills.

## Declarations

### Author contribution statement

Lehulu Tilahun Yirdaw: Conceived and designed the experiments; Analyzed and interpreted the data.

Birhanu Dessu Teferra: Performed the experiments.

Mulusew Zeleke Belay: Analyzed and interpreted the data.

Kirubel Dagnaw Tegegne and Ms Infant Rani Augustin: Contributed reagents, materials, analysis tools or data.

### Funding statement

This work was supported by Wollo University.

### Data availability statement

Data will be made available on request.

### Declaration of interest’s statement

The authors declare no conflict of interest.

### Additional information

No additional information is available for this paper.
